# Clinical differential analysis of severe complications thrombotic microangiopathy and acute graft-versus-host disease following allogeneic hematopoietic stem cell transplantation

**DOI:** 10.3389/fonc.2025.1668243

**Published:** 2025-12-08

**Authors:** Yuan-yuan Wang, Gang-ping Li, Shu-zhen Fu, Yue-wen Fu, Yong Shen, Qing-xia Xu

**Affiliations:** 1 Department of Clinical Laboratory, The Affiliated Cancer Hospital of Zhengzhou University & Henan Cancer Hospital, Zhengzhou, China; 2 Department of Hematology, The Affiliated Cancer Hospital of Zhengzhou University & Henan Cancer Hospital, Zhengzhou, China

**Keywords:** clinical differential analysis, severe complications, thrombotic microangiopathy, acute graft-versus-host disease, allogeneic hematopoietic stem cell transplantation

## Abstract

**Background:**

Identifying the early markers of transplant-associated thrombotic microangiopathy (TA-TMA) and mild or severe acute graft-versus-host disease poses a significant challenge in the differential diagnosis of severe complications after allo-hematopoietic stem cell transplantation (allo-HSCT).

**Methods:**

We conducted an analysis of 109 patients who developed TA-TMA, grade I-II aGVHD , and grade III-IV aGVHD following allo-HSCTs at our center between 2019 and 2024. Clinical features, laboratory data and outcomes were compared among these groups.

**Results:**

The median diagnostic time for TA-TMA was 94 days post-transplant, with median survival of 1 month for TA-TMA and 2 months for grade III-IV aGVHD, significantly shorter than 5 months for grade I-II aGVHD. Survival curves for TA-TMA and grade III-IV aGVHD were poorer. Risk factors (RFs) for TA-TMA included fragmented red blood cell proportion, elevated LDH, and renal injury, with hemoglobin showing a protective effect. Cox analysis identified hypertension, cardiac insufficiency, renal injury, fragmented red blood cells, LDH, platelet counts, hemoglobin, and albumin as significant mortality factors for TA-TMA. For grade III-IV aGVHD, hypertension, renal injury, fragmented red blood cells, LDH, TBIL, platelet counts, hemoglobin, and albumin were significant. Multivariate analysis showed that fragmented red blood cells, elevated LDH, and renal injury predicted higher mortality for TA-TMA, while higher platelet counts and albumin levels predicted lower mortality. No independent prognostic factors were found for grade III-IV aGVHD.

**Conclusion:**

Our analysis highlights the significant challenge of late diagnosis and high mortality in TA-TMA following allo-HSCT. It further identifies a panel of accessible clinical indicators that can aid in early risk assessment and prognostication.

## Introduction

Transplant-associated thrombotic microangiopathy (TA-TMA) and acute graft-versa-host disease (aGVHD) are typically manifesting within the first 100 days post-transplant. These conditions represent critical factors influencing the success of HSCT, with aGVHD being the most prevalent, but TA-TMA poses a greater threat due to its rapid progression and high mortality rate (1–7). Diagnosis is usually based on the criteria stipulated by either Europe (European Bone Marrow Transplant International Working Group) (8) or United States (Blood and Marrow Transplantation Clinical Trial Network) (9), but the uniformity of these standards remains to be studied. Grade 2–4 aGVHD was found to be an adjusted risk factor for TA-TMA (10) and lead to an increased risk of death in TA-TMA (11), but further research is needed to understand the overlap of disease mechanisms and the underlying relationships. aGVHD usually occurs in the acute phase after allo-HSCT, when TA-TMA is most commonly observed (5). Both TA-TMA and grade III-IV aGVHD exhibit numerous overlapping clinical symptoms, making it more difficult to distinguish between these two diseases. To date, there have been no reports on distinguishing TA-TMA from aGVHD, an accurate diagnosis and early start of etiological treatment that alter the natural history of these serious conditions may best improve patient outcomes.

## Materials and methods

### Patient selection/data source

This study was conducted at the Hematology Department of Henan Cancer Hospital, and this trial was registered with the Chinese Clinical Trial Registry (SBGJ202102069). This retrospective study consecutively enrolled 376 patients who underwent allo-HSCT at our institution between January 2019 and June 2024. Among them, 38 patients (10.1%) developed TA-TMA, 153 (40.7%) developed grade I–II aGVHD, and 64 (17.0%) developed grade III–IV aGVHD. Patients in the TA-TMA cohort were excluded if they met any of the following criteria: (1) missing essential clinical or laboratory data at the time of diagnosis; (2) co-occurrence of other severe thrombotic disorders, such as disseminated intravascular coagulation or heparin-induced thrombocytopenia; or (3) death within 21 days after transplantation, which precluded adequate assessment of complications. 33 patients with TA-TMA were included in the final analysis. For comparative purposes, 51 patients with grade I–II aGVHD and 25 with grade III–IV aGVHD were randomly selected using matching ratios ranging from 1:2 to 1:3. Matching was based on age (± 3 years), transplantation modality, and human leukocyte antigen (HLA) compatibility. The exclusion criteria for patients with aGVHD are the same as those for TA-TMA. The information collected includes primary diseases (including acute myeloid leukemia [AML], acute lymphoblastic leukemia [ALL], myelodysplastic syndrome [MDS], severe aplastic anemia [SAA]), other hematological diseases [others]), clinical course records, clinical symptoms, laboratory test results, and patient follow-up information.

### TA-TMA, GVHD definitions and managements

The diagnostic criteria for TA-TMA can meet any of the following established standards: the BMT-CTN criteria (9), the O-TMA criteria (12), the Probable-TA-TMA criteria (12), or the Jodele criteria (13, 14). Acute GVHD was graded and staged using Glucksberg criteria, and grading of cGVHD was performed according to Seattle criteria (15). And the concordance among patients meeting different TA-TMA criteria was shown in **Supplementary Table 1**.

### Data collection and definition

Routine laboratory tests included complete blood count, urine routine, liver function, renal function, myocardial enzymes, c-reactive protein (CRP), and the fragmented red blood cell proportion in peripheral blood. The virus serological status was determined to be either negative or positive based on the quantitative results of cytomegalovirus (CMV), Epstein-Barr virus (EBV), and polyomavirus BK (PBK) before HSCT and at the time of diagnosis. Transplant-related factors included transplant type, stem cell sources, ABO blood type compatibility between donor and recipient, disease status before HSCT, minimal residual disease (MRD) status before HSCT, number of mononuclear cells (MNC) received, number of CD34+ cells received, pretreatment protocol.

### Treatment, outcome and follow-up

Conditioning regimens were selected based on disease status, patient age, and comorbidities. The primary myeloablative conditioning (MAC) regimens were busulfan/cyclophosphamide (Bu/Cy) and cyclophosphamide/total body irradiation (Cy/TBI). The main reduced-intensity conditioning (RIC) regimens were fludarabine/busulfan (Flu/Bu) and fludarabine/melphalan (Flu/Mel). For GVHD prophylaxis, most patients with matched related or unrelated donors received a calcineurin inhibitor (tacrolimus) plus methotrexate. Effective management of TA-TMA is hampered by obscure pathogenesis and delayed diagnosis. There are no well-acknowledged therapeutic strategies for TA-TMA. The effective management of TA-TMA is hindered by an elusive pathogenesis and often delayed diagnosis. Currently, no universally standardized therapy exists. Management primarily centers on complement-targeted agents (eculizumab) and comprehensive supportive care to address complications such as hypertension and renal dysfunction. While interventions like therapeutic plasma exchange (TPE), rituximab, or defibrotide may be considered in specific cases, they lack robust evidence and are not routinely recommended. Follow-up was conducted by telephone until December 31, 2024, including efficacy of treatment, patient outcomes and survival status.

### Statistical analysis

Differences between groups were compared using the Mann-Whitney *U* test for continuous variables and the chi-squared test or Fisher’s exact test for categorical variables. The probabilities of overall survival (OS) was estimated using Kaplan-Meier method and the groups were compared using log-rank test. Multivariate analysis of potential confounders associated with diagnosis was conducted using Cox proportional hazards regression model, both univariate and multivariate analyses of key factors associated with diagnosis and prognosis were performed using Cox proportional hazards regression model, with hazard ratios (HR) and 95% confidence intervals (95% CI) reported. *p* value <0.05 was considered significant. Data were processed and analyzed using SPSS Version 27.0 (SPSS, Inc) and R version 4.0.3 (R Foundation for Statistical Computing, Vienna, Austria).

## Results

### Clinical features of patients with TA-TMA and aGVHD

The demographic and clinical characteristics of the included cases are shown in **Table 1**. Our data showed no obvious gender tendency (*p*>0.05) and no significant age concentration (*p*>0.05) in the disease groups, and there were no significant differences in the type of primary disease, the pre-transplant MRD positivity rate, and the pre-transplant disease remission status (all *p*>0.05; **Table 1**). Among the 33 patients diagnosed with TA-TMA, 21.2% (7/33) developed the disease within one month post allo-HSCT, 57.6% (19/33) within three months post allo-HSCT, and 97.0% (32/33) within one year post allo-HSCT. The median time post-transplant diagnosis of TA-TMA, grade I-II aGVHD, and grade III-IV aGVHD was 94 days (range 39 to 667 days), 30 days (range 6 to 136 days), and 28 days (range 6 to 184 days), respectively, with TA-TMA being the latest (*p* < 0.001). 31 TA-TMA patients were combined with aGVHD (one of which was late aGVHD, and five were converted from aGVHD to cGVHD), one was combined with cGVHD, and one had no GVHD. All TA-TMA diagnosis were during GVHD process.

**Table 1:** Clinical features of patients.

Characteristics	TA-TMA (N=33)	Grade I-II aGVHD (N=51)	Grade III-IV aGVHD (N=25)	*P*
Male gender, *n* (%)	20 (60.6)	30 (58.8)	18 (72.0)	0.521
Age at diagnosis, years, Mean (SD)	24.6 (14.5)	32.3 (14.9)	29.9 (16.0)	0.078
Primary disease, *n* (%)				0.264
AML	6 (18.2)	15 (29.4)	8 (32.0)	
ALL	10 (30.3)	14 (27.5)	10 (40.0)	
MDS	8 (24.2)	5 (9.8)	3 (12.0)	
SAA	7 (21.2)	9 (17.6)	4 (16.0)	
Others	2 (6.1)	8 (15.7)	0 (0.0)	
Transplant method, *n* (%)				0.554
Non-blood	11 (33.3)	16 (31.4)	8 (32.0)	
Sibling fully matched	4 (12.1)	14 (27.5)	6 (24.0)	
Haploid	18 (54.5)	21 (41.2)	11 (44.0)	
Stem cell sources, *n* (%)				0.667
Bone marrow	0 (0.0)	0 (0.0)	1 (4.0)	
Peripheral blood	32 (97.0)	48 (94.1)	23 (92.0)	
Bone marrow+Peripheral blood	1 (3.0)	3 (5.9)	1 (4.0)	
Pretreatment protocol, *n* (%)				0.352
MAC	16 (48.5)	24 (47.1)	16(64)	
RIC	17 (51.5)	27(52.9)	9 (36)	
Disease status before HSCT, *n* (%)^a^				0.252
CR	16 (64.0)	36 (85.7)	18 (85.7)	
PR or NR	9 (36.0)	6 (14.3)	3 (14.3)	
MRD before HSCT, *n* (%)^a,b^				
Positive	9 (34.6)	7 (16.7)	5 (23.8)	0.273
Negative	17 (65.4)	35 (83.3)	16 (76.2)	
CMV-emia before HSCT, *n* (%)	1 (3.0)	0 (0.0)	1 (4.0)	0.274
EBV-emia before HSCT, *n* (%)	2 (6.1)	3 (5.9)	0 (0)	0.586
PBK-emia before HSCT, *n* (%)	3 (9.1)	3 (5.9)	0 (0)	0.407
Cord blood assisted transplantation, *n* (%)	6 (18.2)	6 (11.8)	6 (24.0)	0.374
ABO compatibility, *n* (%)	20 (60.6)	33 (64.7)	14 (56.0)	0.759
First transplantation, *n* (%)	31 (93.9)	50 (98)	23 (92.0)	0.426
MNC count,×10^8^/Kg, Mean (SD)	14.9 (6.8)	13.6 (6.8)	10.9 (4.9)	0.067
CD34^+^ cells count,×10^6^/Kg, Mean (SD)	8.2 (5.6)	6.3 (3.4)	5.7 (3.0)	0.055
granulocyte implantation time, days, Mean (SD)	12.7 (1.7)	12.2 (2.7)	13.0 (2.0)	0.285

AML,acute myelocytic leukemia;ALL,acute lymphocytic leukemia; MDS,myelodysplastic; syndrome; SAA, severe aplastic anemia; MAC, myeloablative conditioning; RIC, reduced-intensity conditioning; CR, complete remission; PR, partial remission; NR, non-remission; MNC, mononuclear cell count; MRD; minimal residual disease; CMV, cytomegalovirus; EBV, EB virus; PBK, polyomavirus BK.

a Indicates that patients with n=20 SAA were excluded from the MRD and disease status statistics before HSCT; MRD was measured by flow cytometry before HSCT and MRD levels≥10^−4^ was defined as positive

### Analysis of transplant-related factors and laboratory data of three groups

Transplant-related results showed that there were no significant differences among the groups in first transplantation or not, transplantation method, stem cell sources, with or without cord blood assisted transplantation, ABO compatibility, pretreatment protocol, incidence of CMV-emia, EBV-emia, and PBK-emia before HSCT, CD34+ cells count, MNC count, and granulocyte implantation time post-transplantation (all *p*>0.05; **Table 1**). Three disease groups were prone to intestinal infection, EBV-emia, PBK-emia, and positive urinary protein at the onset of disease, and there was no significant difference in symptoms (all *p*>0.05). There were no significant differences among the three groups in numbers of white blood cells, AST, CREA, and CRP levels (all *p*>0.05). The incidence of hypertension, cardiac insufficiency, renal injury, pulmonary infection, CMV-emia and serum LDH and BUN levels in TA-TMA group were significantly higher than those in grade I-II aGVHD and grade III-IV aGVHD groups, and the counts of red blood cells and hemoglobin levels were significantly lower in the TA-TMA group (all *p* < 0.05). The incidence of positive urinary occult blood and hemorrhagic cystitis in TA-TMA and grade III-IV aGVHD groups were higher than those in aGVHD I-II group, and the levels of platelet and serum ALB in TA-TMA and grade III-IV aGVHD groups were lower than those in grade I-II aGVHD group. Serum TBIL levels in III-IV aGVHD group were lower than those in TA-TMA and grade I-II aGVHD groups (all *p* < 0.05 or *p* = 0.05; **Table 2**).

**Table 2:** Laboratory data and combined clinical symptoms of patients at the time of onset.

Variables at onset of illness	TA-TMA (N=33)	Grade I-II aGVHD (N=51)	Grade III-IV aGVHD (N=25)	*P*
Hypertension, *n* (%)	12 (36.4)	6 (11.8)	6 (24.0)	0.028
Cardiac insufficiency, *n* (%)	18 (54.5)	12 (23.5)	2 (8.0)	< 0.001
Renal injury, *n* (%)	15 (45.5)	5 (9.8)	3 (12.0)	< 0.001
Pulmonary infection, *n* (%)	30 (90.9)	32 (62.7)	19 (76.0)	0.015
Intestinal infection, *n* (%)	20 (60.6)	24 (47.1)	14 (56.0)	0.454
Hemorrhagic cystitis, *n* (%)	14 (42.4)	8 (15.7)	9 (36.0)	0.019
CMV-emia, *n* (%)	13 (39.4)	7 (13.7)	4 (16.0)	0.015
EBV-emia, *n* (%)	7 (21.2)	7 (13.7)	4 (16.0)	0.637
PBK-emia, *n* (%)	13 (39.4)	12 (23.5)	12 (48.0)	0.078
Positive urinary protein, *n* (%)	10 (30.3)	11 (21.6)	7 (28.0)	0.640
Positive urinary occult blood, *n* (%)	13 (39.4)	6 (11.8)	8 (32.0)	0.010
White blood cells,×10^9^/L, Median (IQR)	2.3 (1.1, 3.9)	2.5 (1.3, 4.7)	2.2 (1.0, 4.4)	0.899
Red blood cells, ×10^12^/L, Mean(SD)	2.4 (0.6)	2.8 (0.4)	2.9 (0.9)	0.004
Hemoglobin, g/L, Mean (SD)	77.8 (16.4)	91.8 (18.2)	90.2 (27.4)	0.007
Platelets,×10^9^/L, Median (IQR)	28.0 (15.0, 52.0)	56.0 (27.5, 82.0)	28.0 (21.0, 41.0)	0.007
Serum ALB, g/L, Mean (SD)	35.7 (5.8)	39.9 (5.5)	35.4 (5.5)	< 0.001
Serum TBIL, μmol/L, Median (IQR)	17.8 (11.4, 38.4)	14.6 (10.6, 24.0)	33.7 (12.1, 50.7)	0.050
Serum AST, U/L, Median (IQR)	45.0 (26.0, 68.0)	26.0 (18.0, 52.0)	39.0 (20.0, 60.0)	0.080
Serum LDH,U/L, Median (IQR)	687.0 (471.0, 1000.0)	287.0 (252.5, 413.0)	342.0 (249.0, 506.0)	< 0.001
Serum BUN, mmol/L, Median (IQR)	9.0 (5.8, 14.2)	5.3 (3.4, 9.2)	6.6 (3.5, 11.4)	0.003
Serum CREA, μmol/L, Median (IQR)	71.0 (29.0, 106.0)	49.0 (36.5, 62.5)	47.0 (36.0, 73.0)	0.336
Serum CRP, mg/L,Median (IQR)	20.0 (1.5, 36.0)	5.8 (1.0, 37.3)	20.6 (2.6, 36.0)	0.488

CMV, cytomegalovirus; EBV, EB virus; PBK, polyomavirus BK; ALB, albumin; TBIL, total bilirubin; AST, aspartate aminotransferase; LDH, lactate dehydrogenase; BUN, blood urea nitrogen; CREA, creatinine; CRP, C-reactive protein.

### Fragmented red blood cells in peripheral blood

**Figure 1:**
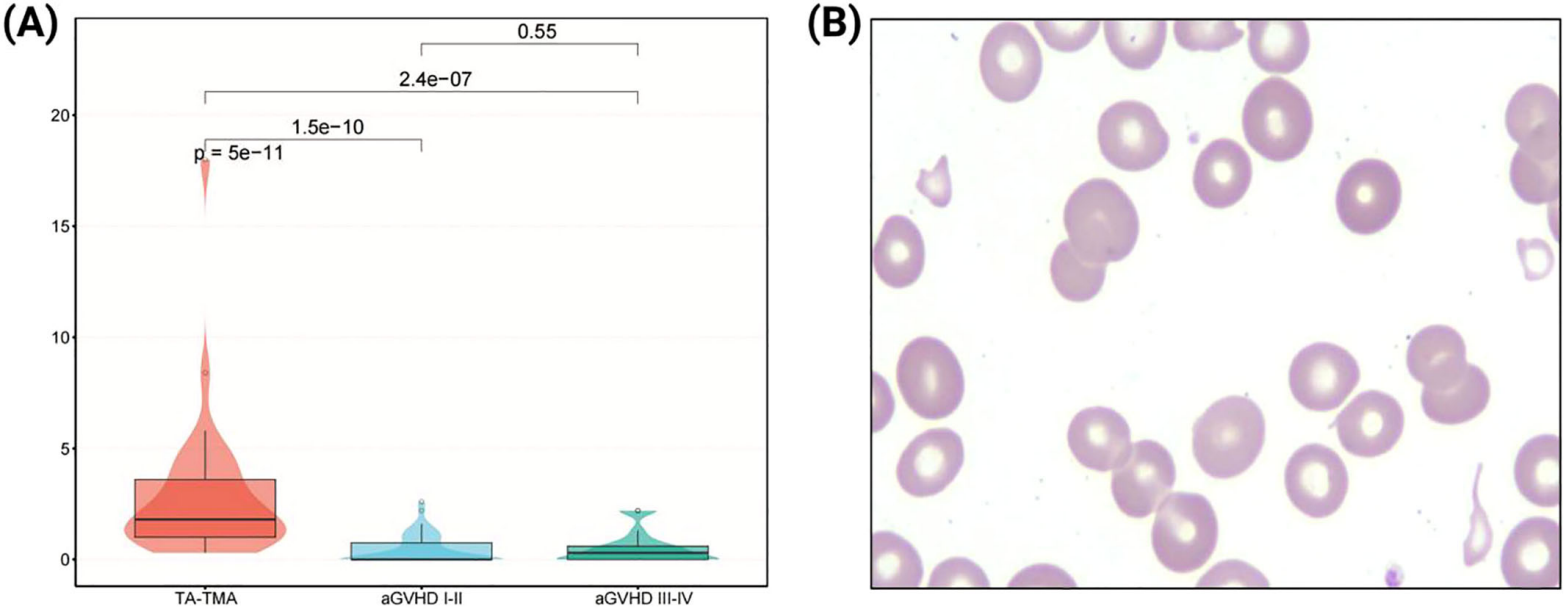
Analysis of fragmented red blood cells in peripheral blood. **(A)** Comparison of the proportion of fragmented red blood cells among three groups. **(B)** Morphological features of fragmented red blood cells in peripheral blood smear (1000×).

At the time of diagnosis, the proportion of fragmented red blood cells in peripheral blood of TA-TMA patients ranged from 0.6% to 18%, with a Median (IQR) of 1.8% (1.0%, 3.6%), of which only 6 cases (18.2%) were <1%, and 16 cases (48.5%) were between 1% and 3%, 11 cases (33.3%) were ≥3%. Meanwhile, in patients with grade I-II aGVHD, the proportion ranged from 0.0% to 2.6%, with a Median (IQR) of 0.0% (0.0%, 0.8%), of which 39 cases (76.5%) were <1%, and the rest were <3%. In patients with grade III-IV aGVHD, the proportion ranged from 0.0% to 2.2%, with a median (IQR) of 0.3% (0.0%, 0.6%), of which 21 cases (84.0%) were <1%, and the rest were <3%. Proportion of fragmented red blood cells in peripheral blood in TA-TMA patients was significantly higher (*p* < 0.001; **Figure 1**).

### Analysis of patients’ survival

**Figure 2:**
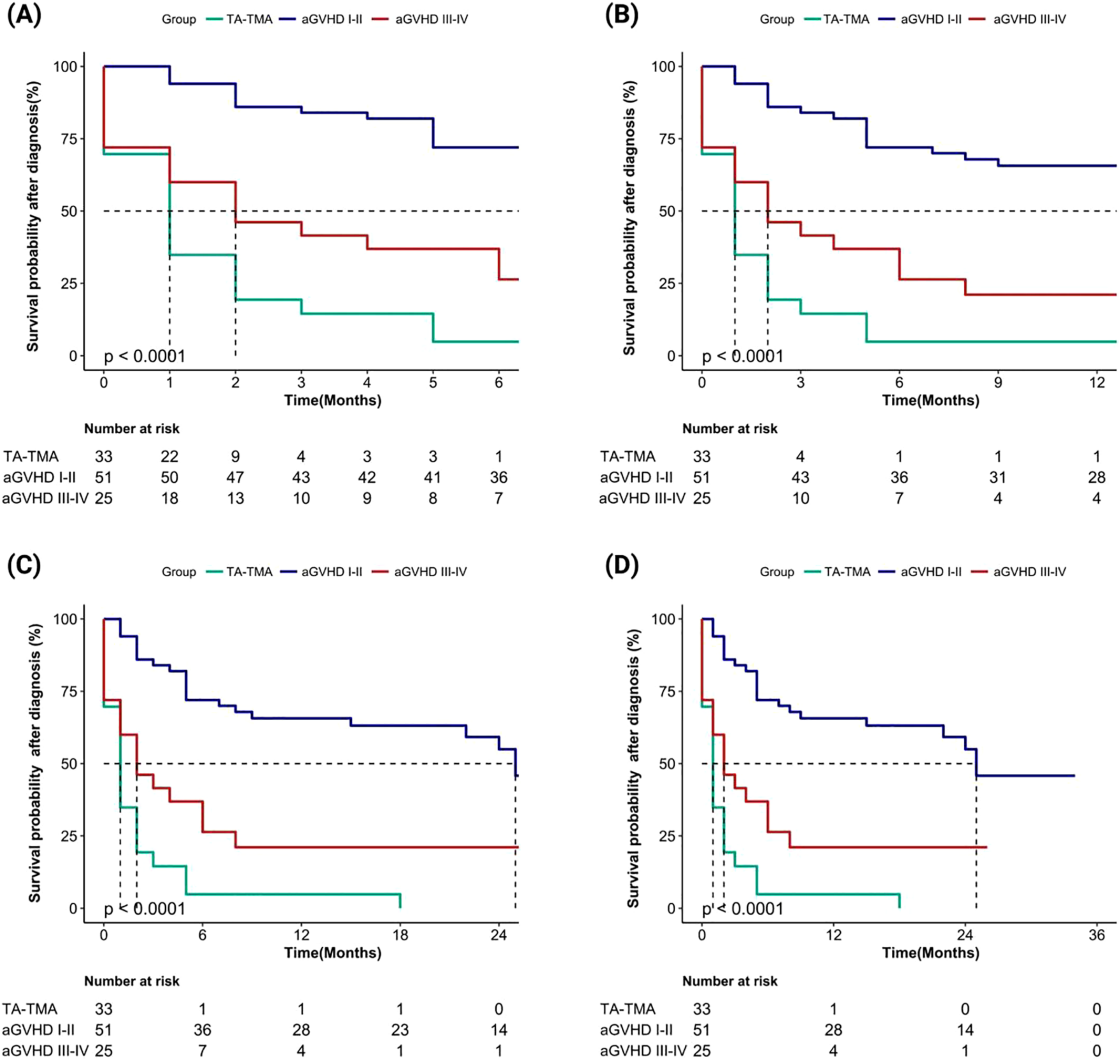
Comparison of overall survival rates after diagnosis among TA-TMA, aGVHD I-II and aGVHD III-IV groups (aGVHD I-II: grade I-II aGVHD; aGVHD III-IV: grade III-IV aGVHD ). **(A)** Patients’ survival at 6 months; **(B)** Patients’ survival at 1 year; **(C)** Patients’ survival at 2 years; **(D)** Patients’ survival at 3 years.

**Figure 3:**
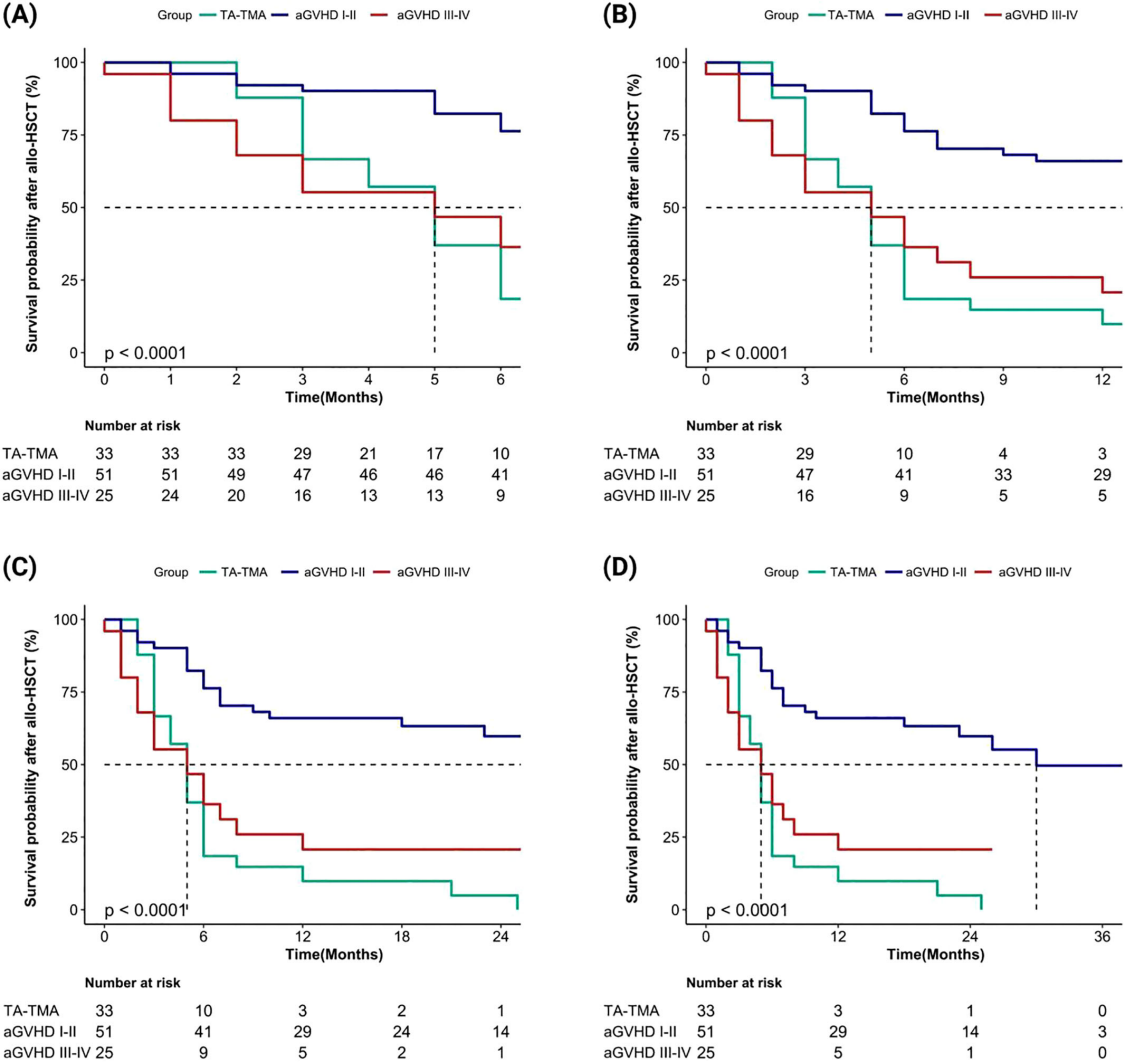
Comparison of overall survival rates after allo-HSCT among TA-TMA, aGVHD I-II and aGVHD III-IV groups (aGVHD I-II: grade I-II aGVHD; aGVHD III-IV: grade III-IV aGVHD). **(A)** Patients’ survival at 6 months; **(B)** Patients’ survival at 1 year; **(C)** Patients’ survival at 2 years; **(D)** Patients’ survival at 3 years.

We evaluated the cumulative mortality and overall survival of 109 patients up to the follow-up date. The cumulative mortality rates for TA-TMA, grade I-II aGVHD, and grade III-IV aGVHD were 90.9% (30/33), 43.1% (22/51), and 72.0% (18/25), respectively. The interquartile range (IQR) of overall survival months after allo-HSCT were 5.0 (3.0, 6.0), 16.0 (7.0, 26.0), and 5.0 (2.0, 7.0), respectively. The IQR of the overall survival months after diagnosis were 1.0 (0.0, 2.0), 15.0 (5.0, 24.0), and 2.0 (0.0, 6.0), respectively. Compared to grade I-II aGVHD, the mortality rates of TA-TMA and grade III-IV aGVHD were significantly higher, and both survival months after allo-HSCT and after diagnosis were significantly worse (all *p* < 0.001). We further calculated the overall survival rates of patients at 6 months, 1 year, 2 years, and 3 years after allo-HSCT and after diagnosis, the results showed that the overall survival rate of TA-TMA was the worst, followed by grade III-IV aGVHD and grade I-II aGVHD, and the differences among the three groups were statistically significant (all *p<*0.001; **Figure 2**, **Figure 3**).

### Multivariate analysis for the occurrence of disease

To further understand the risk factors for disease occurrence, we performed multivariate Cox regression analysis between groups separately. Risk assessment results, with outcome variable set as TA-TMA and grade I-II aGVHD showed that an increased proportion of fragmented red blood cells in peripheral blood (per 1% increase, HR 0.25, 95% CI 0.10-0.62, *p* = 0.003), serum LDH levels (per 1 unit increase of transformed ln, HR 0.05, 95% CI 0.10-0.31, *p* = 0.001), and renal injury (HR 0.08, 95% CI 0.01-0.45, *p* = 0.004) were significantly associated with the risk of developing TA-TMA (**Table 3**). For outcome variables set as TA-TMA and grade III-IV aGVHD, the risk assessment revealed that an increased proportion of fragmented red blood cells in peripheral blood (per 1% increase, HR 0.22, 95% CI 0.08-0.61, *p* = 0.003), hemoglobin levels (per 1 unit increase of transformed ln, HR 2.72, 95% CI 1.00-7.40, *p* = 0.050), serum LDH levels (per 1 unit increase of transformed ln, HR 0.09, 95% CI 0.02-0.49, *p* = 0.006), and renal injury (HR 0.08, 95% CI 0.01-0.53, *p* = 0.009) were significantly associated with the risk of developing TA-TMA, among which hemoglobin was a protective factor for TA-TMA (**Table 3**).

**Table 3:** Risk factors for the occurrence of patients with TA-TMA, grade I-II and III-IV aGVHD.

Variables	Grade I-II aGVHD *vs.*TA-TMA			Grade III-IV aGVHD *vs.*TA-TMA		
	HR	95%CI	*P*	HR	95%CI	*P*
Fragmented red blood cells, per 1% increase	0.25	0.10-0.62	0.003	0.22	0.08- 0.61	0.003
CMV-emia	1.33	0.64-3.03	0.441	1.42	0.45- 2.28	0.715
Renal injury	0.08	0.01~0.45	0.004	0.08	0.01~ 0.53	0.009
Platelets, per 10×10^9^ increase	1.40	0.64-3.03	0.396	1.02	0.45- 2.28	0.968
Hemoglobin, (ln), per 1 unit increase	1.78	0.70~4.56	0.226	2.72	1.00~ 7.40	0.050
LDH (ln), per 1 unit increase	0.05	0.01~0.31	0.001	0.09	0.02~ 0.49	0.006
ALB (ln), per 1 unit increase	2.48	0.17~36.70	0.509	0.06	0.00~ 1.37	0.078

CMV,cytomegalovirus; LDH, lactate dehydrogenase; ALB, albumin.

### Risk factors for death in TA-TMA

The median overall survival of TA-TMA after allo-HSCT and diagnosis was only 5 months and 1 month, respectively, although treatment measures were taken immediately after diagnosis, there was no significant improvement in the survival rate of TA-TMA. The leading causes of death were infection, active TA-TMA, and multiple organ dysfunction syndrome (MODS). Therefore, we introduced variables into COX regression model, with death for all causes as the competing risk. Univariate analysis showed that CMV-emia and hemorrhagic cystitis did not increase the risk of death from TA-TMA (both *p*>0.05), in contrast, several factors were found to be significantly associated with the risk of death from TA-TMA: hypertension (HR 2.01, 95%CI 1.19-3.39, *p* = 0.009), cardiac insufficiency (HR 1.79, 95%CI 1.10-2.92, *p* = 0.019), renal injury (HR 2.94, 95%CI 1.70-5.08, *p* < 0.001), proportion of fragmented red blood cells in peripheral blood (per 1% increase, HR 2.94, 95%CI 1.78-4.85, *p* < 0.001), LDH levels (per 1 unit increase of transformed ln, HR 2.59, 95%CI 1.75-3.83, *p* < 0.001), platelet counts (per 10×10^9^ increase, HR 0.99, 95%CI 0.98-1.00, *p* = 0.005), hemoglobin levels (per 1 unit increase of transformed ln, HR 0.16, 95%CI 0.05-0.52, *p* = 0.002), and ALB levels (per 1 unit increase of transformed ln, HR 0.10, 95%CI 0.02-0.43, *p* = 0.002) (**Table 4**). Cox multivariate analysis showed that a higher proportion of fragmented red blood cells in peripheral blood (per 1% increase, HR 1.84, 95%CI 1.03-3.31, *p* = 0.040), elevated serum LDH levels (per 1 unit increase of transformed ln, HR 1.67, 95%CI 1.07-2.62, *p* = 0.025) and renal injury (HR 2.42, 95%CI 1.25-4.69, *p* = 0.009) were prone to be independent risk factors associated with an increased risk of death for TA-TMA, conversely, higher platelet counts (per 10×10^9^ increase, HR 0.99, 95%CI 0.99-1.00, *p* = 0.043) and elevated serum ALB levels (per 1 unit increase of transformed ln, HR 0.20, 95%CI 0.04-0.97, *p* = 0.046) were prone to be independent protective factors associated with a decreased risk of death for TA-TMA (**Table 4**).

**Table 4:** Univariate and multivariate Cox regression analysis affecting the death of TA-TMA.

Variables	Univariate analysis			Multivariate analysis		
	HR	95%CI	*P*	HR	95%CI	*P*
Female sex	0.88	0.66~1.45	0.560	–	–	–
Age, per 10 years	1.37	0.96~2.12	0.096	–	–	–
Fragmented red blood cells, per 1% increase	2.94	1.78~4.85	<0.001	1.84	1.03~3.31	0.040
CMV-emia	1.51	0.89~2.56	0.129	–	–	–
Hypertension	2.01	1.19~3.39	0.009	1.18	0.61~2.27	0.622
Cardiac insufficiency	1.79	1.10~2.92	0.019	0.91	0.50~1.64	0.752
Renal injury	2.94	1.70~5.08	<0.001	2.42	1.25~4.69	0.009
Hemorrhagic cystitis	1.40	0.83~2.37	0.213	–	–	–
Platelets, per 10×10^9^ increase	0.99	0.98~1.00	0.005	0.99	0.99~1.00	0.043
Hemoglobin (ln), per 1 unit increase	0.16	0.05~0.52	0.002	0.70	0.18~2.67	0.599
LDH (ln), per 1 unit increase	2.59	1.75~3.83	<0.001	1.67	1.07~2.62	0.025
ALB (ln), per 1 unit increase	0.10	0.02~0.43	0.002	0.20	0.04~0.97	0.046

CMV,cytomegalovirus; LDH, lactate dehydrogenase; ALB, albumin.

### Risk factors for death in grade III-IV aGVHD

The survival curve for patients with grade III-IV aGVHD was inferior only to that of TA-TMA. By analyzing univariate Cox proportional risk ratios, it was determined that hypertension (HR 1.80, 95%CI 1.07~3.03, *p* = 0.028), renal injury (HR 1.97, 95%CI 1.15~3.37, *p* = 0.013), proportion of fragmented red blood cells in peripheral blood (transformed-ln, HR 1.81, 95%CI 1.28~2.57, *p* = 0.001), TBIL levels (per 1 unit increase of transformed-ln, HR 1.01, 95%CI 1.00~1.01, *p* = 0.006), platelet counts (per 10×10^9^ increase, HR 0.91, 95%CI 0.85~0.98, *p* = 0.008), hemoglobin levels (per 1 unit increase of transformed-ln, HR 0.23, 95%CI 0.07~0.70, *p* = 0.010), ALB levels (per 1 unit increase of transformed-ln, HR 0.19, 95%CI 0.05~0.73, *p* = 0.015) jointly affected the survival of grade III-IV aGVHD patients. Cox multivariate analysis showed that none of the above factors exhibited independent clinical prognostic significance (all *p*>0.05; **Table 5**).

**Table 5:** Univariate Cox regression analysis affecting the death of grade III-IV aGVHD.

Variables	Univariate analysis			Multivariate analysis		
	HR	95%CI	*P*	HR	95%CI	*P*
Female sex	0.84	0.51~1.37	0.486	–	–	–
Age, per 10 years	1.00	0.98~1.02	0.933	–	–	–
Fragmented red blood cells, per 1% increase	1.13	1.05~1.21	0.002	1.11	0.99~1.25	0.066
CMV-emia	1.55	0.91~2.63	0.108	–	–	–
Hypertension	1.80	1.07~3.03	0.028	1.41	0.69~2.88	0.341
Cardiac insufficiency	1.49	0.91~2.44	0.112	–	–	–
Renal injury	1.97	1.15~3.37	0.013	1.31	0.68~2.54	0.423
Hemorrhagic cystitis	1.52	0.89~2.57	0.122	–	–	–
Platelets, per 10×10^9^ increase	0.91	0.85~0.98	0.008	0.96	0.89~1.04	0.331
Hemoglobin (ln), per 1 unit increase	0.23	0.07~0.70	0.010	0.58	0.16~2.05	0.396
LDH (ln), per 1 unit increase	1.81	1.28~2.57	0.001	1.53	0.94~2.50	0.087
ALB (ln), per 1 unit increase	0.19	0.05~0.73	0.015	1.11	0.21~5.97	0.905
TBIL(ln), per 1 unit increase	1.01	1.00~1.01	0.006	1.00	1.00~1.01	0.375

CMV, cytomegalovirus; LDH, lactate dehydrogenase; ALB, albumin; TBIL, Total bilirubin.

### Survival outcomes of TA-TMA patients

**Figure 4:**
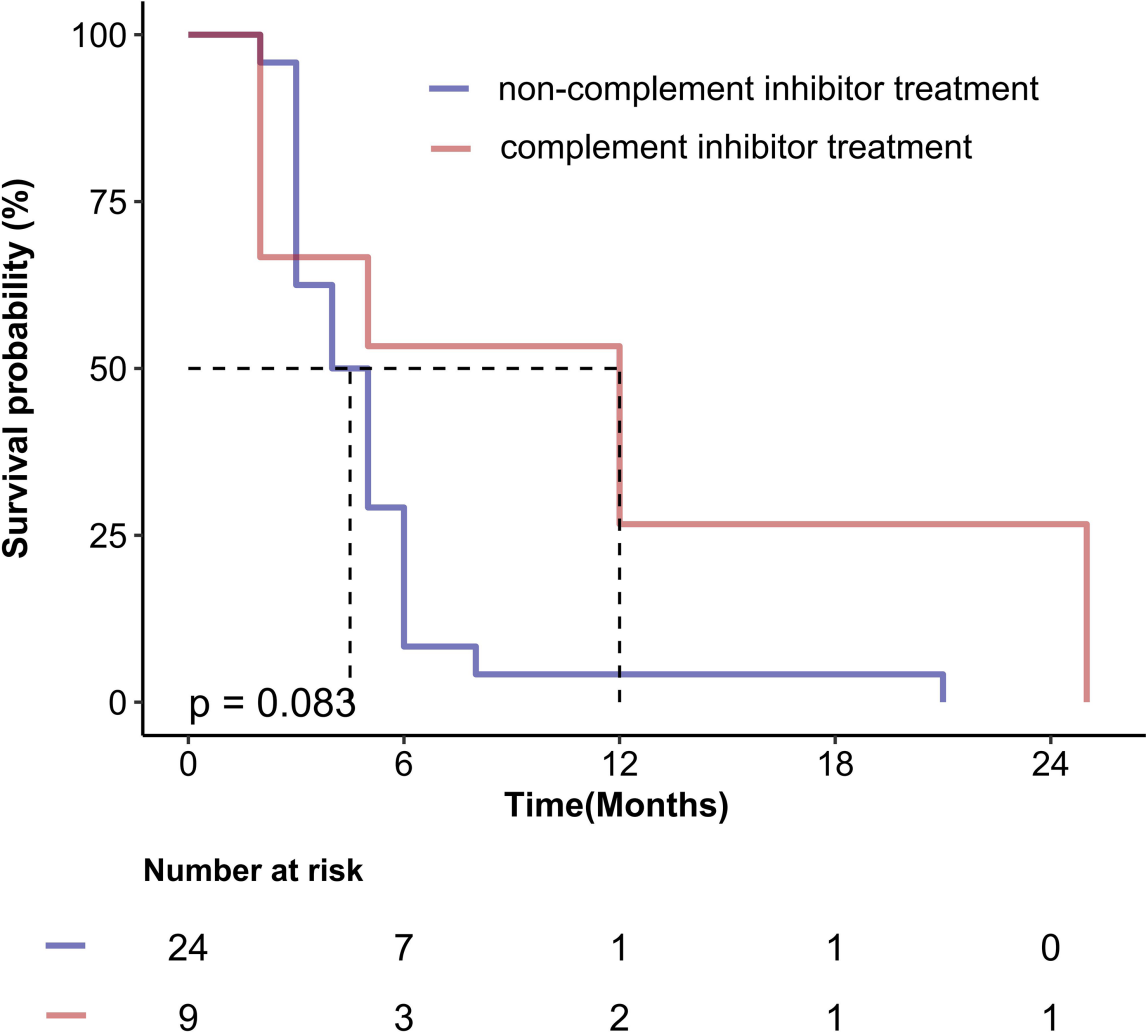
Overall survival of TA-TMA patients according to the therapeutic strategies.

Given the critical importance of treatment strategy, we performed an exploratory comparison of survival outcomes based on the primary therapies received by patients with TA-TMA. Among the 33 patients in the cohort, 9 received complement inhibitor treatment therapy, while the remaining 24 received only supportive care for complications and/or therapeutic plasma exchange. It is noteworthy that all three surviving patients were treated with eculizumab, although no statistically significant difference was observed between the groups (*p* = 0.083). The corresponding Kaplan-Meier survival curve is presented in **Figure 4**.

## Discussion

TA-TMA is a microangiopathic hemolytic anemia and platelet consumption resulting from complement-mediated endothelial injury following allo-HSCT, resulting in microvascular thrombosis and fibrin deposition in the microcirculation (16). aGVHD, another more common and early complication after allo-HSCT, is induced by the direct endothelial injury mediated by host-reactive, donor-derived cytotoxic T cells (5). TA-TMA and grade 2–4 aGVHD are recognized risk factors for mortality after allo-HSCT (5, 17, 18), the clinical differentiation of the two represents a diagnostic challenge for transplant clinicians. All TA-TMAs in this study occurred during the process of GVHD. In our study, the cumulative mortality rate of TA-TMA was 90.9%, while the cumulative mortality rate of grade III-IV aGVHD was also as high as 72.0%, both of which are serious complications after allo-HSCT. Most studies have shown that the occurrence time of TA-TMA is between 32 days and 303 days after HSCT (5, 13, 17–25). In our study, the median onset time of TA-TMA was the 94th day after allo-HSCT, which was later than that of aGVHD, indicating that the occurrence of TA-TMA is more insidious, and may have a slow onset but a dangerous development.

The impact of various transplantation factors on TA-TMA has been reported by multiple institutions, including aGVHD, sex, prior autologous transplant, GVHD prophylaxis, donor type, source of stem cells (25, 26). And factors related to the risk of aGVHD are mainly reported as age and donor type (27–29). Our results showed no obvious tendency in age and gender between TA-TMA and aGVHD, nor were there any notable differences in the transplant-related factors examined in this study. To date, no literature has concurrently compared and analyzed these factors in both TA-TMA and aGVHD, precluding reference to differing outcomes. It is therefore worthy to incorporate a broader range of transplant-related indicators in future multi-center and long-term studies.

In 2012, the International Council for Standardization in Hematology (ICSH) proposed that more than 1% of morphologically identified schistocytes on the blood film are considered suspicious for TMA (30). Report for 2021 suggested that the detection of more than 1% schistocytes on PB smears in adults and full-term neonates remained a robust cytomorphological threshold that favors a diagnosis of TMA (31). In our study, the proportion of fragmented red blood cells in TA-TMA was more than 1%, which was consistent with the literature reports. The proportion of fragmented red blood cells in TA-TMA was significantly higher compared to those in aGVHD, and the decrease in the proportion of these fragmented cells can reduce the risk of TA-TMA. The increased proportion of fragmented red blood cells elevated the risk of death of TA-TMA by 80%, which can independently predict TA-TMA, highlighting its close association with the occurrence, development and prognosis of TA-TMA. On the other hand, it is worth noting that although all TA-TMA patients in this study consistently exhibited fragmented red blood cells in peripheral blood, a lack such cells does not exclude *a priori* the diagnosis of TMA, it had been reported that the absence of fragmented red blood cells in patient with TA-TMA had led to a delay diagnosis and treatment (32), emphasizing the importance of fragmented red blood cells combined with specific laboratory tests and comprehensive analysis.

Not only that, the injury to vascular endothelial cells in TA-TMA patients may lead to microangiopathic hemolytic anemia and thrombocytopenia, as well as thrombosis in the microcirculation (33, 34). These adverse effects may subsequently cause ischemic damage to end-organs, particularly the kidneys, as well as the lungs, heart, and gastrointestinal tract. In contrast, the initial phase of aGVHD is characterized by tissue damage mediated through the regulation and release of inflammatory cytokines, primarily affecting three areas: the skin, gastrointestinal tract, and liver (35). Based on the different pathogenesis and the analysis results of this study, we identified five symptoms that are more likely to co-occur with TA-TMA: hypertension, renal injury, cardiac insufficiency, pulmonary infection, and CMV-emia. Additionally, three significantly elevated indicators in TA-TMA patients were identified: proportion of fragmented red blood cells in peripheral blood, serum LDH levels, and serum BUN levels. Two significantly reduced indicators were also noted: red blood cell counts and hemoglobin levels. For grade III-IV aGVHD, TBIL levels were higher and associated with a higher risk of death, and increased serum TBIL was a unique marker of hepatic involvement (35), suggesting that patients with grades III-IV aGVHD are more likely to liver involvement. Although TA-TMA was associated with a higher incidence of hemorrhagic cystitis and urinary occult blood and a reduction in platelet and ALB levels, these indicators were similarly observed in our grade III-IV aGVHD patients, therefore, hemorrhagic cystitis, urinary occult blood, platelets counts, and ALB levels are more significant only when differentiating between TA-TMA and grade I-II aGVHD cases.

Existing studies have reported varying evidence of the prognostic impact of LDH and renal injury in TA-TMA. High baseline LDH level was associated with definite and probable TMA, definite and probable TMA were each independently associated with long-term kidney dysfunction (19), elevated LDH levels, proteinuria on routine urinalysis, and hypertension were the earliest markers of TMA (13). HSCT-TMA was associated with renal dysfunction, renal failure, renal replacement therapy and hypertension (36). Our findings determined that LDH elevation and renal injury were more likely significantly associated with an increased risk of TA-TMA. On this basis, we also noticed that elevated LDH, and renal injury predicted higher mortality for TA-TMA, representing one of the novel findings of this study. This finding is consistent with recent literature indicating that the ASTCT/CIBMTR/EBMT/APBMT consensus risk stratification for pediatric TA-TMA identifies LDH >2 times ULN is one of the most important predictors of nonrelapse mortality of TA-TMA, allowing risk stratification even in the absence of available sC5b-9 testing (37). Although proteinuria is closely related to TA-TMA, our study did not reveal a significant difference in the incidence of proteinuria between TA-TMA and aGVHD, so no further analysis was conducted, considering that proteinuria frequently occurs when aGVHD involves the kidneys. These data suggest that more transplant recipients should be included in our subsequent studies and clinical trials to verify the discriminatory and prognostic value of proteinuria between these two diseases.

A pre-emptive strategy has successfully decreased the incidence of CMV disease after allo-HSCT, however, it is difficult to completely prevent that (38). Positive CMV serostatus is also associated with probable TMA (19), which may act as an independent risk factor for TA-TMA and directly damage microvascular endothelial cells, causing TMA (13, 14). Kraft S’s research showed that CMV reactivation/end-organ disease was a risk factor for development of TA-TMA (5), only a limited number of studies suggested that the risk factors for TA-TMA were not associated with CMV-emia (28). In this study, no significant difference was observed in the CMV positivity rate between TA-TMA and aGVHD prior to transplantation. However, at the time of diagnosis, the CMV positivity rate of TA-TMA was significantly higher than that of aGVHD. Compared to grade I-II and III-IV aGVHD, post-transplant CMV viremia may increase the risk of TA-TMA development and death, although the result was not statistically significant after adjusting for risk variables. Regardless, post-transplant CMV viremia should not be ignored, while considering aGVHD after transplantation, clinicians should be more vigilant for the development of TA-TMA.

Hemoglobin levels, platelet counts, ALB levels were identified as protective factors for the occurrence, development, and prognosis of TA-TMA in our study. Severe anemia and severe thrombocytopenia had been recognized as significant prognostic indicators in a large-scale external validation trial of the TA-TMA prognostic model (39), and our findings are in agreement with the existing literature reports. Furthermore, we found that the levels of hemoglobin and platelets in TA-TMA were significantly lower than those in aGVHD, which has certain clinical value for the differential diagnosis between TA-TMA and aGVHD, and increased hemoglobin and platelet levels can reduce the risk of death for TA-TMA and grade III-IV aGVHD, serving as protective factors against the death for TA-TMA and grade III-IV aGVHD. Notably, platelet levels retained independent prognostic significance for TA-TMA even after adjusting for various risk variables. Although platelets are often at low levels due to the insufficiency of megakaryocyte engraftment in the early stages after allo-HSCT, the onset of TA-TMA typically occurs later than that of aGVHD, so we speculate that it is not difficult to exclude the interference of low platelet levels in the differential diagnosis of TA-TMA and aGVHD at this time, despite the current inability to fully evaluate this hypothesis. Furthermore early post-allo-HSCT studies are warranted to validate the applicability of this finding in contemporary patient cohorts. In addition, ALB has been identified as an independent prognostic factor following allo-HSCT in leukemia patients, capable of predicting long-term outcomes (40). In this study, ALB levels in TA-TMA and grade III-IV aGVHD were significantly lower compared to those in grade I-II aGVHD, and was significantly associated with a reduced risk of mortality in TA-TMA, acting as an independent predictor of favorable prognosis for TA-TMA. Patients with TA-TMA may consume ALB through urinary albumin loss and inflammation. The underlying mechanism is primarily associated with kidney lesions and inflammation resulting from microvascular endothelial injury, while ALB depletion in aGVHD is mainly attributed to protein loss caused by gastrointestinal injury, so in clinical practice, dynamic monitoring of ALB levels should be integrated with other biomarkers for a comprehensive evaluation. Despite limitations of sample size and retrospective design, our exploratory analysis revealed a non-significant trend toward improved survival with complement inhibitor in TA-TMA. Recent studies have demonstrated that complement inhibitors can markedly enhance survival outcomes in both adult and pediatric patients with TA-TMA (3, 41), suggesting a potential survival benefit for high-risk individuals. Prospective studies are urgently needed to definitively establish the efficacy of targeting the complement pathway in high-risk TA-TMA.

To our knowledge, this study provides one of the first comprehensive analyses aimed at differentiating TA-TMA from aGVHD following transplantation. Our findings suggest that fragmented red blood cells, renal injury, and serum LDH levels may serve as independent diagnostic indicators for distinguishing TA-TMA from aGVHD. Furthermore, hemoglobin levels appear to have diagnostic utility in differentiating TA-TMA from severe (grade III-IV) aGVHD. In terms of prognosis, our data provide preliminary evidence that fragmented red blood cells, LDH, and renal injury are associated with an increased risk of mortality, whereas platelet counts and ALB levels may be potential protective factors in TA-TMA patients.

There are some limitations in this study, due to the limited sample size and the large range of screened variables, there is no further analysis of all potentially significant and important indicators. An important aspect of our study design was the comparative analysis of TA-TMA and aGVHD as mutually exclusive groups, despite the established fact that aGVHD is a significant risk factor for TA-TMA development. Our rationale for this approach was pragmatic and aimed at addressing a specific clinical question: at the moment of initial clinical deterioration, which parameters can most effectively help the clinician distinguish whether the primary driver is aGVHD or TA-TMA? We sought to delineate the relatively “pure” clinical profiles of each entity at onset. We acknowledge that this design is a simplification and does not capture the complexity of cases where both entities manifest simultaneously or sequentially. Future dynamic studies should build upon to unravel their complex interplay. In addition, the small sample size and single-center design limit the statistical power and generalizability of our findings. The prognostic outcomes of TA-TMA in this study, particularly the independent prognostic factors identified through multivariate analysis-including schistocytes, lactate dehydrogenase, renal injury, platelets, and albumin should be regarded as exploratory preliminary results. These findings require validation in larger sample sizes and should not be considered definitive independent prognostic factors. The primary value of this study lies in its detailed descriptions and the provision of valuable real-world insights, which offer robust hypotheses for future research.

Based on our findings, we recommend a rapid TA-TMA diagnostic approach using key lab parameters (Hb, fragmented RBCs, LDH, renal injury), alongside monitoring platelets and albumin for risk stratification and renal protection. Future studies should investigate the protective roles of these factors, assess early interventions, and validate the survival benefit of complement blockade in high-risk patients.

## Data Availability

The original contributions presented in the study are included in the article/**Supplementary Material**. Further inquiries can be directed to the corresponding author.
