# Supplementary material for: Clinical differential analysis of severe complications thrombotic microangiopathy and acute graft-versus-host disease following allogeneic hematopoietic stem cell transplantation
Source: Front Oncol. 2025 Dec 8;15:1668243. doi: 10.3389/fonc.2025.1668243 (PMC12719282; doi:10.3389/fonc.2025.1668243)
Supplement: Supplementary file 1 [file Table1.docx]

Supplementary Table 1. The situation of 33 TA-TMA patients meeting different diagnostic criteria

| Patients | BMT-CTN criteria | O-TMA criteria | Probable-TA-TMA criteria | Jodele criteria |
| --- | --- | --- | --- | --- |
| Patient 1 | No | Yes | Yes | No |
| Patient 2 | No | Yes | Yes | Probable |
| Patient 3 | Yes | Yes | Yes | No |
| Patient 4 | No | Yes | Yes | No |
| Patient 5 | No | Yes | Yes | No |
| Patient 6 | No | Yes | Yes | No |
| Patient 7 | No | Yes | Yes | Yes |
| Patient 8 | No | Yes | Yes | No |
| Patient 9 | No | Yes | Yes | No |
| Patient 10 | Yes | Yes | Yes | No |
| Patient 11 | Yes | Yes | Yes | No |
| Patient 12 | No | Yes | No | No |
| Patient 13 | No | Yes | Yes | No |
| Patient 14 | No | Yes | Yes | Probable |
| Patient 15 | No | Yes | Yes | No |
| Patient 16 | No | Yes | Yes | No |
| Patient 17 | Yes | Yes | Yes | No |
| Patient 18 | No | Yes | Yes | No |
| Patient 19 | No | Yes | Yes | No |
| Patient 20 | No | Yes | Yes | No |
| Patient 21 | No | Yes | Yes | No |
| Patient 22 | No | Yes | Yes | No |
| Patient 23 | No | Yes | Yes | No |
| Patient 24 | No | Yes | Yes | No |
| Patient 25 | No | Yes | Yes | No |
| Patient 26 | No | Yes | Yes | No |
| Patient 27 | No | Yes | No | No |
| Patient 28 | No | Yes | Yes | No |
| Patient 29 | No | Yes | Yes | No |
| Patient 30 | Yes | Yes | Yes | No |
| Patient 31 | No | Yes | Yes | No |
| Patient 32 | No | Yes | Yes | Yes |
| Patient 33 | No | Yes | Yes | Probable |
| In total (n, %) | 5 (15.2%) | 33 (100%) | **31 (93.9%)** | 1 (3.0%) |
